# Modification and Clinical Application of the Inner Perivitelline Membrane Test in Different Avian Species

**DOI:** 10.3390/vetsci6020039

**Published:** 2019-04-12

**Authors:** Judith Krohn, Dominik Fischer, Helena Schneider, Klaus Failing, Michael Lierz, Christine Ehling, Axel Wehrend

**Affiliations:** 1 Clinic for Obstetrics, Gynecology and Andrology of Large and Small Animals with Ambulatory Service, Faculty of Veterinary Medicine, Justus-Liebig-University, Frankfurter Str. 106, 35392 Giessen, Germany; axel.wehrend@vetmed.uni-giessen.de; 2 Clinic for Birds, Reptiles, Amphibians and Fish, Faculty of Veterinary Medicine, Justus Liebig University, Frankfurter Str. 91, 35392 Giessen, Germany; dominik.fischer@vetmed.uni-giessen.de (D.F.); helena.schneider@vetmed.uni-giessen.de (H.S.); michael.lierz@vetmed.uni-giessen.de (M.L.); 3 Unit for Biomathematics and Data Processing, Faculty of Veterinary Medicine, Justus Liebig University, Frankfurter Str. 95, 35392 Giessen, Germany; Klaus.Failing@vetmed.uni-giessen.de; 4 Institute for Farm Animal Genetics of the Friedrich-Löffler-Institute, Höltystr. 10, 31535 Neustadt, Germany; christine.ehling@gmx.de

**Keywords:** functional sperm-assay, assisted reproduction, perivitelline-membrane-penetration, bird

## Abstract

The aim of this study was to adapt an inner perivitelline membrane (IPVM) test as an interspecies penetration assay for avian spermatozoa. The IPVM of different bird species was evaluated to test the penetrating ability of avian spermatozoa in an intra- and interspecies design. Isolation of the IPVM via acid hydrolysis was tested in pre-incubated chicken eggs and in six other avian species. The separation protocol was modified (time, acid concentration) to facilitate practicability. Separated membranes were evaluated with dark field microscopy for the presence of holes produced by penetrating spermatozoa. In chicken eggs, the influence of different membrane storage conditions was tested. In the penetration assay, the IPVM of chicken eggs was used as a model for fresh and frozen–thawed rooster sperm and for fresh spermatozoa of cockatiels and falcons. Results demonstrated that the time of egg-incubation had a significantly negative influence on the isolation ability of the IPVM (*p* < 0.0001). IPVM-separation was successful for a maximum of two days after preincubation. In the experiments with eggs from other avian species, results were heterogenous: there was no isolation in geese and cockatiels, 20% in the European kestrel, and 40% in pheasant, quail, and duck. In the penetration assay, holes were found in 100% of the IPVM of chicken eggs after incubation with native and frozen–thawed rooster semen and in 10% with fresh cockatiel semen. Falcon spermatozoa failed to produce visible holes. In conclusion, the IPVM of chicken eggs seems to be unsuitable to establish a functional sperm assay in other species tested but is suitable for quality evaluation of cryopreserved rooster sperm.

## 1. Introduction

Functional assays for spermatozoa are acknowledged as a valuable method to assess male reproductive ability in andrology. Various studies in infertile men have shown that a normal spermiogram may occur despite severe functional defects of spermatozoa [1]. In avian reproductive medicine, the functional evaluation of spermatozoa is less common, compared to mammalian species. Improvement in assisted-reproductive technologies is important in terms of species conservation [2,3], to reveal causes of infertile clutches and to enable the sorting of infertile males from breeding programs. An interspecific assay would be very useful, especially to test semen of endangered species where eggs of the same species are rarely available. When avian spermatozoa encounter the ovum in the infundibulum of the female reproductive tract, it is surrounded by the inner perivitelline membrane (IPVM) [4]. The IPVM is homologous to the mammalian zona pellucida (ZP). When mammalian spermatozoa bind to the ZP3-glycoprotein, the acrosome reaction of the sperm cell and cortical degranulation of the ZP are induced. The enzymes released by the bound sperm lead to local disintegration of the ZP. Likewise, avian spermatozoa pass through the IPVM by hydrolysis to reach the ovum [4]. After penetration of spermatozoa, holes are present in the IPVM [4]. During the passage of the oviduct, the outer perivitelline membrane and the other egg components are produced. In contrast to most mammals, in which only one spermatozoon penetrates the zona pellucida, numerous sperms commonly hydrolyze holes into the inner perivitelline membrane of avian oocytes (physiological polyspermy) [5]. Determination of sperm penetration can serve as a functional sperm assay [5]. To detect holes of penetrating spermatozoa the inner perivitelline membrane can be extracted from a laid egg. The holes in the IPVM are visible in dark field microscopy after IPVM-isolation, indicating the penetration ability of spermatozoa. These techniques have been used in eggs of domestic chicken (*Gallus gallus domesticus*) [4], duck (*Anas platyrhynchos domestica*) [6], and quail (*Coturnix japonica*) [7], but the applicability for other avian species is unknown. However, a cross reactivity between IPVM of quail, chicken, turkey, and duck with sperm of different species, e.g., turkey, bull, mice, ram, and stallion, has been demonstrated [7,8,9]. Comparable data for psittacines and birds of prey do not exist so far, although they belong to the group of endangered species.

One method of isolation is the incubation of the ovum in hydrochloric acid to separate the inner from the outer perivitelline membrane [5,6,10]. The use of acid hydrolysis to separate the perivitelline membrane of laid eggs is described in the literature [10], but different methodological questions are still to be answered. In the present study, first, the suitability of acid hydrolysis for the isolation of the IPVM in incubated chicken eggs was evaluated. Secondly, it was investigated if age and storage conditions of the egg affects IPVM isolation success. Thirdly, we tested if the separated IPVM of unfertilized chicken eggs can be used as a model for a functional interspecies penetration assay with fresh falcon and cockatiel spermatozoa. Furthermore, the intraspecies IPVM-test was applied for the in vitro evaluation of cryopreserved rooster sperm. 

## 2. Materials and Methods

### 2.1. General Procedures

The acid hydrolysis method, according to Kido and Doi [10], was used to induce the separation of the inner (IPVM) from the outer perivitelline membrane (OPVM). Eggs and fresh semen samples used were provided by the clinic for Birds, Reptiles, Amphibians, and Fish of the Faculty of Veterinary Medicine in Giessen. The keeping and treatment of all animals used during this investigation were carried out in accordance with the European legislation for the protection of animals used for scientific purposes (Directive 2010/63/EU). Preparation of the eggs before IPVM isolation differed in the trials. The yolk with blastoderm was extracted from laid eggs, washed three to four times in 50 mL 0.9% saline solution, and incubated in a hot cabinet at 37 °C in 300 mL of 0.01 mol/L hydrochloric acid (Merck, Darmstadt, Germany) for 60 min. Separation of the inner and outer perivitelline membrane occurred spontaneously during incubation. Subsequently, the yolk membranes were incised using a scalpel blade (OR size 12, Otto Rüttgers GmbH, Solingen, Germany), and the yolk was removed by washing with 1% saline. For this purpose, the membranes were transferred in different beakers containing 50 mL saline, respectively, until the remaining saline was clear. After washing, the membranes were investigated under a stereoscopic microscope (M8, Wild Heerbrugg, Heerbrugg, Switzerland) with twofold magnification. When isolation was successful, the IPVM could be distinguished from the OPVM, being the transparent thin layer of the two existing parts. Sections of the IPVM, 0.5 cm × 0.5 cm in size, were cut out with scissors, spread out onto a slide, and investigated for the presence of holes produced by penetrating spermatozoa, using dark field microscopy [10] (DM R, Leica Microsystems GmbH, Wetzlar, Germany) under 100-fold magnification. If present, the number of holes was counted in ten randomly chosen fields of view and summed up for each section. Beforehand, the slide was roughly evaluated for areas with extremely high or low densities of holes to choose an area with a medium amount for evaluation.

### 2.2. Experiment 1

The aim of this experiment was to test different acid concentrations and incubation times for separation of the IPVM. The protocol reported in previous studies was modified to try to enhance practicability. Chicken (*Gallus gallus domesticus*) eggs up to seven days after laying were used and stored in the refrigerator (4 °C) until investigated. These eggs were potentially fertilized, as hens had access to roosters. Two different hydrochloric acid concentrations (0.01 mol/L, 0.1 mol/L) and incubation times (60 min, 30 min) were tested, and the IPVM isolation success was evaluated (group I: 0.01 mol/L, 60 min, group II: 0.01 mol/L, 30 min, group III: 0.1 mol/L, 60 min, group IV: 0.1 mol/L, 30 min). A total number of ten eggs per group (I–IV) was used. All eggs were obtained from a breeding flock (breed: leghorn, age: 6–12 months).

### 2.3. Experiment 2

The aim of this experiment was to test the effect of hatching on the separation ability by acid hydrolysis. The method of acid hydrolysis was applied to chicken (*Gallus gallus domesticus*) eggs after different periods in an incubator. Thus, fertilized (*n* = 42) and unfertilized (*n* = 42) chicken eggs, stored for up to seven days after laying, were incubated at 37 °C and 55% humidity according to standard procedures (Schumacher Brutmaschine VFG-1, 1973) [11]. Starting on the first day of incubation, six eggs per day were prepared and evaluated every 24 h using a 0.01 mol/L hydrochloric acid and a 60-min incubation time. A daily investigation was performed for seven days. Separated pieces of the IPVM were assessed microscopically, as described above.

### 2.4. Experiment 3

The aim of this trial was to investigate the influence of different storing conditions (time, light exposure, temperature) on the applicability of acid hydrolysis. Unfertilized chicken eggs were collected during a period of five days after laying and having been stored under the following conditions (Table 1): group A, at room temperature (21 °C) and under daylight exposure in the side room of the laboratory (21 °C); group B, in a refrigerator (4 °C) and in darkness; and group C, at room temperature (21 °C) and darkness in the side room of the laboratory. Ten eggs were used per group. The acid hydrolysis was performed during a period of sixteen days in three- to five-day intervals.

### 2.5. Experiment 4

The aim was to test the application of acid hydrolysis in eggs of other avian species. Eggs of the following species were included: cockatiel (*Nymphicus hollandicus*), common quail (*Coturnix coturnix*), European kestrel (*Falco tinnunculus*), goose (*Anser anser domestica*), pheasant (*Phasianus colchicus*), and runner duck (*Anas platyrhynchos domestica*). According to availability, the eggs were freshly collected (duck, quail, goose, cockatiel) or stored in the refrigerator for up to two weeks prior to investigation (pheasant, European kestrel). Upon receipt, all investigated eggs were stored at 4 °C in the refrigerator and prepared after a maximum of five days, according to the results of Experiment 1 (0.01 mol/L hydrochloric acid, incubation 60 min). The number of ten eggs per species was used, except in the pheasant, where only nine eggs were available.

### 2.6. Experiment 5

The aim of the last experiment was testing the IPVM from chicken eggs in an intra- and interspecific penetration assay. The IPVM was extracted from unfertilized chicken eggs. The eggs were collected on the day of laying, and the IPVM was isolated no longer than five days later. The IPVM was either used freshly extracted or stored in saline solution at 4 °C for up to three days. To test the penetration ability of spermatozoa, 0.5 cm × 0.5 cm pieces of chicken IPVM were placed in a tube (Eppendorf tube 3810X, 1.5 mL, Eppendorf AG, Hamburg, Germany) containing 1 mL Dulbecco’s Modified Eagle’s medium (DMEM) (Sigma-Aldrich Chemie GmbH, Taufkirchen, Germany) buffered with 1 mol/L HEPES (N-2-Hydroxyethylpiperazine-N′-2-ethanesulfonic acid) (Sigma-Aldrich Chemie GmbH, Taufkirchen, Germany) respectively. Between 1 × 10^5^ and 2.4 × 10^6^ spermatozoa (variation due to volume and concentration of ejaculate) was added in a volume of 3–5 µL to the IPVM without any preincubation. The semen samples used consisted of fresh-diluted and frozen–thawed cock spermatozoa (groups 1 and 2, as well as fresh and diluted cockatiel spermatozoa (groups 3 and 4) and fresh and diluted falcon spermatozoa (groups 5 and 6)). Thawing of the straws was performed by placing them in iced water (1 °C) for a period of 60 s. Afterwards, 2 µL thawed semen was added to the IPVM in the Eppendorf tube. Subsequently, the tube was incubated in a water bath at 41 °C for ten minutes [6]. Afterward, the piece of IPVM was spread out on a slide and evaluated in dark field microscopy, as described above. Ten pieces of IPVM were evaluated with each ejaculate.

The fresh samples were collected using the abdominal massage method [12,13,14,15,16]. Prior to incubation, semen analysis was performed, including the examination of volume and color, density and progressive motility, pH, concentration, and live-dead ratio in combination with sperm morphology [17]. All samples were used within three hours after semen collection. Cock spermatozoa were diluted 1:2, and falcon spermatozoa were diluted at a 1:1 ratio with a commercially available diluent (6-Hour SemAid, PHL Associates, Inc., Davis, CA, USA) at 4 °C. The cockatiel spermatozoa were diluted at a ratio of 1:1, up to 1:3 depending on the concentration with a specific diluent for cockatiels [18]. Diluted samples were stored in the refrigerator (4 °C) before use. Each ejaculate was divided into five (falcon/cockatiel) or ten (cock) fractions and incubated with pieces of IPVM.

The cryopreserved rooster semen was obtained from the Friedrich–Löeffler-Institute of Farm Animal Genetics in Mariensee. The frozen cock spermatozoa had been collected by abdominal massage and sperm of ten roosters had been pooled. The cooling and freezing process followed the protocol of Ehling, et al. [19]. The fresh ejaculate pool was first diluted with HS1-medium (1:1 ratio) [20], and, second, with N-methylacetamid and dimethylformamid (2:1 ratio) at 4 °C. The final diluted samples were packaged in 0.25 mL straws (300 million sperm per straw) and cooled with −3 °C steps/min down to −35 °C and then with −50 °C/min down to −130 °C. The straws were stored in liquid nitrogen. Overall motility and progressive motility had been assessed by CASA (CASA-System, Hamilton Thorne, Beverly, MA, USA), and morphology was assessed microscopically before and after freezing.

The number of holes in the IPVM was counted in ten randomly chosen fields of view. In slides with more than 300 holes per field of view, no individual count was performed, and in the results were indicated as >300 holes per field of view. 

For statistical analyses, the program packages BMDP/Dynamic, Release 8.1 [21], and LogXact-Turbo [22] were used. Data was evaluated using an exact multi-factorial logistic regression model to test for the influence of incubation time, acid concentration, storage conditions, and fertilization on successful isolation. The difference between bird species was examined with the Fisher’s exact test. For evaluation of the relationship between sperm concentration and number of holes, a regression analysis was done and additionally, Spearman’s rank correlation coefficient was calculated. The level of significance for each test done was α = 0.05.

## 3. Results

### 3.1. Experiment 1

Modification of the incubation protocol from Kido and Doi [10] did not result in a better isolation of the IPVM (Table 2). 

**Figure 1:**
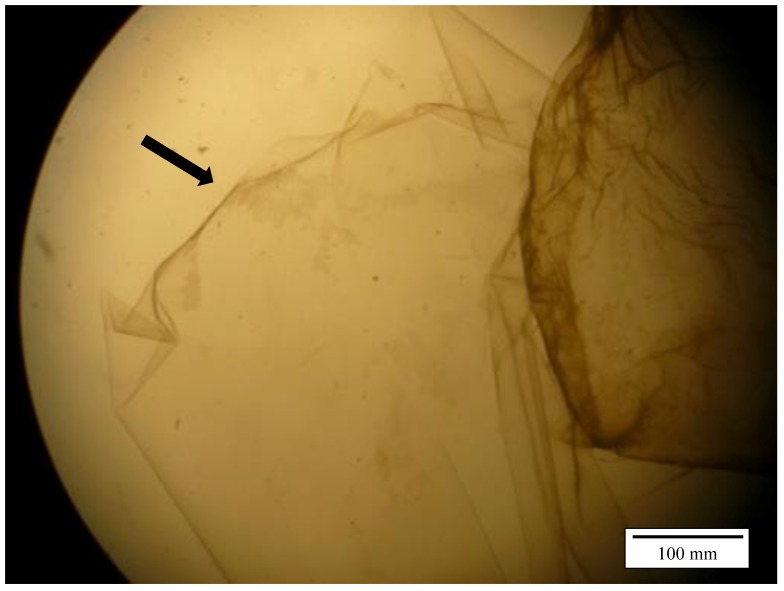
Separated perivitelline membrane of a chicken egg under the stereo microscope (x2): Arrow indicates inner part.

There was no significant difference between group I + III vs. II + IV (30 and 60 min incubation, 0.01 mol/L HCl) (*p* = 0.582). Between the two different acid concentrations (group I + II vs. III + IV) the difference was highly significant (*p* < 0.0001) with successful separation in 9 out of 10 cases using 0.01 mol/L HCl. Figure 1 illustrates the separated inner and outer part of the perivitelline membrane. During the trial with 0.1 mol/L hydrochloric acid, no isolation of the IPVM was possible. 

### 3.2. Experiment 2

In unfertilized eggs, the isolation of the inner and outer perivitelline membrane was possible until two days (48 h) in the incubator. The shorter the eggs were incubated before application of acid hydrolysis, the better the results that were achieved. After an incubation time over 48 h the isolation of the IPVM was not successful.

In potentially fertilized eggs, the isolation of the IPVM was successful on day one (100%) and on day two (16.6%). However, no separation was detected on day three. From day four onward, the developing embryo inhibited the yolk isolation. Additionally, all three eggs from the potentially fertilized group being discovered without a developing embryo at day six of incubation were evaluated; however, in none of these eggs was the isolation of inner and outer perivitelline membrane successful. The only parameter found to have a significant influence on the isolation ability was the time of incubation (*p* < 0.0001). Fertilization did not have a statistically significant influence (*p* = 0.12). 

### 3.3. Experiment 3

The results of the influence of storage parameters on isolation success are summarized in Table 3. 

From all tested storage parameters, only time had a significant influence (*p* = 0.010) on the IPVM separation in chicken eggs. None of the other parameters was significant, and there was no reciprocal effect.

### 3.4. Experiment 4

The isolation success of the IPVM in other avian species is summarized in Table 4.

No isolation was achieved in eggs of goose and cockatiel. Using kestrel eggs, the isolation was successful in 20% (2 out of 10) of eggs and in, at least, 40% (4 out of 10) of eggs in pheasant, quail, and runner duck. Fisher’s exact test pointed out that the difference between species was statistically significant (*p* = 0.012). 

### 3.5. Experiment 5

**Figure 2:**
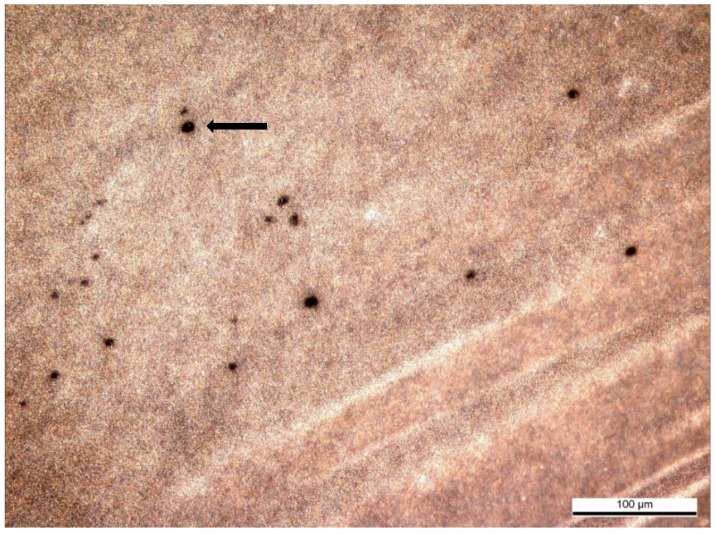
Holes in the chicken IPVM produced by penetrating frozen–thawed cock spermatozoa (×100).

The IPVM penetration assay with cock spermatozoa confirmed the function of the experimental design. Penetration ability of spermatozoa was evaluated by holes in the IPVM with fresh and frozen–thawed cock spermatozoa (Figure 2). 

The relationship between the sperm concentration (fresh sperm/cock) and median number of holes was statistically significant (r_s_ = 0.720, *p* = 0.0125). The number of holes found in the pieces of perivitelline membrane incubated with fresh and frozen–thawed cock spermatozoa are shown in Table 5. 

In the trial with frozen–thawed cock spermatozoa, the number of holes found in the IPVM was highest in comparison to all other investigated ejaculates. 

In the trial with cockatiel spermatozoa, holes were produced in 10% (1 out of 10) of samples. No holes were found after incubation with falcon sperm.

## 4. Discussion

The interaction between the IPVM and spermatozoa can be tested in vitro and thus serves as a functional sperm assay [4,6,7,8,23]. The detection of perivitelline-membrane-bound sperm with fluorescence microscopy is another option to verify the penetration ability of spermatozoa and indicate germ-cell-compatibility. This technique avoids the problem of separating different parts of the perivitelline membrane, as no separation is necessary. Results in various species, including birds [9,23,24,25], chelonians [26], and crocodiles [27], prove this test to be highly useful. 

Different physiological events are important with respect to sperm-egg interaction, including: first of all, transport to the site of fertilization; second, binding of spermatozoa to the perivitelline membrane; and third, penetration. Penetration assays have been developed for different species: in-vitro cervical mucus penetration assay for human [28], bovine [29], and equine spermatozoa [30]. In most assays, synthetically produced substitutes are used to replace the natural penetration medium. For avian species, a similar test has been established: penetration of a dense solution of Accudenz^®^ (Accurate Chemical & Scientific Corporation, Westbury, NY, USA) is used to determine the progressive motility of sperm [31] and is correlated to fertility in the chicken [32]. King et al. [33] were able to prove a correlation between the sperm mobility test and distinct CASA (computer assisted semen analysis)-parameters in turkeys (*Melleagris gallopavo*). However, all these tests contribute only to determining the ability of gametes to reach the site of fertilization. The penetration of the perivitelline membrane needs to be evaluated in another way. In this context, the IPVM-penetration-assay can give valuable information. Similar to human oocytes, unfertilized eggs from rare or endangered species (falcon/psittacine) are difficult to obtain and, if available, only in small numbers for laboratory analysis. Therefore, the IPVM of chicken eggs was intended to serve as a model for a functional penetration assay for other avian species using falcons and psittacines as examples in the conducted trials. 

Although the IPVM-penetration-assay was described in the literature, different methodological questions were still open, and an answer to these questions was the aim of the present study.

In the first experiment, we tried to modify the acid hydrolysis protocol described in the literature to save preparation time by increasing the acid concentration. This modification did not show success, which is why the original protocol was used for the following experiments.

The results in the literature and our own investigation show that isolation of the inner perivitelline membrane via acid hydrolysis is possible in various species from laid eggs. For this reason, we tried to adapt this method to incubated eggs in the second experiment. If this was successful, an incubated, but undeveloping, egg could be evaluated for penetration of spermatozoa. According to our results, this is not a suitable method for evaluation of the perivitelline membrane in incubated eggs due to severe problems with separating inner and outer parts of the perivitelline membrane. A potential alternative is described by Birkhead et al. [34], who used fluorescent dye to visualize sperm nuclei and holes produced by penetrating spermatozoa in incubated eggs of the zebra finch (*Taeniopygia guttata*) and the tree sparrow (*Passer montanus*), collected after the incubation. They were able to evaluate full-term incubated eggs without separating the inner from the outer perivitelline membrane. Therefore, this seems to be a more promising procedure, which may also be applied in exotic and free-ranging bird populations and in eggs with unknown times of incubation.

The storage of eggs after laying can have a potential impact on the success of separation of the IPVM. This aspect has so far not been investigated systematically. As separation of incubated eggs did not work with the applied method, storage was tested in unincubated chicken eggs in the third experiment. Results indicate that the duration of storage has the most influence and therefore a storage time as short as possible is recommended.

In the interspecies comparison (experiment 4), we were able to show that the acid hydrolysis is applicable in eggs of different bird species. Separation of the IPVM was proven in four other species. No separation was found in eggs from cockatiel and goose. Poor separation (20%) in the eggs of the European kestrel can be explained by the fact that for investigation of this species, only preincubated eggs were available.

In the fifth experiment, the IPVM-penetration-assay was performed in an intra- and interspecies design. The interaction of IPVM with fresh and frozen–thawed cock spermatozoa indicated a successful technical design of the conducted trials. Surprisingly, the number of penetrating spermatozoa was greater in the investigation with frozen–thawed spermatozoa, which indicates better motility. In general, cryopreservation reduces the quality of an ejaculate [35,36], and there are species-specific differences in susceptibility to cryopreservation [20,23]. The difference in quality may be due to different reasons: the roosters used for collection of the fresh samples had also been allowed to perform natural service intermittent to the investigation. Time between taking the ejaculates varied, which also can affect the sperm quality [37]. The roosters kept as donors for production of frozen semen were exclusively held for this purpose and, therefore, better trained for and used to the procedures. Moreover, the sperm concentration was higher in the cryopreserved ejaculates due to pooling of high-quality samples. Furthermore, the evaluation of the ejaculates was performed before incubation with the pieces of IPVM. Interactions between components of diluent and incubation media (DMEM) could be responsible for differences in motility between fresh–diluted and frozen–thawed ejaculates.

A cross reactivity between the IPVM and spermatozoa of different species has been reported by various authors [8,9,10]. In contrast, the hen’s IPVM did not interact appropriately with the spermatozoa of cockatiels and falcons in the present study. A possible explanation could be the phylogenetic distance between the species. Win et al. [6] investigated the interaction of chicken semen with quail perivitelline membrane, and these species can even produce hybrids with chicken as the male parent. This makes in-vitro binding of sperm to the IPVM expectable.

Future studies could use the IPVM of closer-related species in the same study design to try to achieve better results regarding the penetration ability of spermatozoa from an individual male. In species-conservation breeding programs, the identification of infertile males is very important in order to use potential breeders to an optimum, and to not block females in pairs by infertile males. Therefore, assays to test spermatozoa function to fertilize eggs are very valuable in addition to commonly used spermatological evaluations. However, in those species, eggs are also rare and IPVMs of the same species are usually not available. Consequently, interspecies IPVM-assays would be a great achievement. However, the results of the study demonstrate that this cannot be achieved between every species. The IPVM of chickens cannot be used to evaluate falcon semen and had poor results with cockatiel semen. The fact that both avian families are distinct from chicken, but partial success was nevertheless achieved, shows that several combinations need to be tried to find IPVMs of common species to test rare species. It is presumed that the more closely they are related, the better the chances there are for interspecies’ use of the test. As it is usually possible to find a common-related species to an endangered one, the results of this study are very promising to establish the IPVM-test as a functional test in avian reproduction. It is necessary to adapt the protocol for IPVM isolation for every species and, if the goal is an interspecies assay, to look for a suitable combination.

## Figures and Tables

**Table 1:** Storage conditions tested to influence the separation of the inner perivitelline membrane (IPVM) of unfertilized chicken eggs.

Group	Storage Conditions	No. of Eggs Investigated per Group
A	21 °C, light exposure	10
B	4 °C, darkness	10
C	21 °C, darkness	10

**Table 2:** IPVM isolation success in unfertilized chicken eggs influenced by separation protocol.

Protocol	No. of Eggs	Concentration of HCl in mol/L	Time of Incubation (min)	Separation Successful (%)	Separation Failed (%)
I	10	0.01	60	9 (90)	1 (10)
II	10	0.01	30	7 (70)	3 (30)
III	10	0.1	60	0 (0)	10 (100)
IV	10	0.1	30	0 (0)	10 (100)

**Table 3:** Influence of storage conditions on the success of separation of the IPVM in unfertilized chicken eggs.

Group	Storage Conditions	Separation Successful n (%)	Separation Failed n (%)	Period of Successful Separation/Days
A	21 °C, light exposure	2 (20)	8 (80)	3
B	4 °C, darkness	6 (60)	4 (40)	14
C	21 °C, darkness	3 (30)	7 (70)	7

**Table 4:** Success of IPVM isolation in eggs of other avian species.

Species	No. of Eggs (n)	Separation Successful n (%)	Separation Failed n (%)
European kestrel (*Falco tinnunculus*)	10	2 (20)	8 (80)
Pheasant (*Phasianus colchicus*)	9	4 (44)	5 (56)
Runner duck (*Anas platyrhynchos domestica*)	10	5 (50)	5 (50)
Quail (*Coturnix coturnix*)	10	4 (40)	6 (60)
Goose (*Anser anser domestica*)	10	0 (0)	10 (100)
Cockatiel (*Nymphicus hollandicus*)	10	0 (0)	10 (100)

**Table 5:** Results of the IPVM penetration assay with fresh and frozen–thawed cock spermatozoa (n = pieces of IPVM) are shown below.

Ejaculate No.	Fresh Semen: No. of Holes (*n* = 10)	Frozen–Thawed Semen: No. of Holes (*n* = 10)
1	48	1240
2	20	1244
3	239	720
4	212	529
5	31	185
6	33	1142
7	284	1048
8	13	1210
9	1.832	1075
10	219	684
11	1.300	/
